# Effects of Laser Plasma Formation on Quasi-Phase Matching of High-Order Harmonics from Nanoparticles and Atoms

**DOI:** 10.3390/nano9040572

**Published:** 2019-04-08

**Authors:** Rashid A. Ganeev, Ganjaboy S. Boltaev, Vyacheslav V. Kim, Chunlei Guo

**Affiliations:** 1 The Guo China-US Photonics Laboratory, State Key Laboratory of Applied Optics, Changchun Institute of Optics, Fine Mechanics and Physics, Chinese Academy of Sciences, Changchun 130033, China; ganjaboy_boltaev@mail.ru (G.S.B.); mik750594@yahoo.com (V.V.K.); 2 The Institute of Optics, University of Rochester, Rochester, NY 14627, USA

**Keywords:** nanoparticles, microparticles, quasi-phase matching, high-order harmonic generation, laser-produced plasma

## Abstract

The application of nanoparticles (NPs) and quasi-phase matching (QPM) each play an important role in the enhancement of high-order harmonics (HHG) of ultrashort laser pulses. We analyze various regimes of nanoparticle plasma formation for the creation conditions for maximal QPM-induced enhancement of the groups of harmonics in the extreme ultraviolet (XUV). Laser plasmas were formed on the surfaces of NPs- and microparticle (MPs)-contained targets using ablation by nanosecond, picosecond, and femtosecond pulses. Different conditions of laser plasma formation (extended and perforated plasma) and variable concentrations of free electrons in these three cases of laser ablation led to modifications of QPM conditions. We demonstrate novel approaches in the optimization of QPM at the conditions of laser ablation of NPs and MPs by pulses of different durations. The formation of QPM conditions using femtosecond and picosecond heating pulses during HHG in such plasmas allowed the growth of conversion efficiency of the groups of harmonics, with the enhancement factors exceeding 25× in different ranges of XUV, contrary to less efficient QPM in the case of nanosecond pulse-induced ablation.

## 1. Introduction

High-order harmonic generation (HHG) using ultrashort laser pulses remains a most reliable method of formation of the table-top coherent extreme ultraviolet (XUV) sources possessing unique temporal characteristics. The practical applications of such sources of radiation require the amendment of HHG efficiency and the enhancement of single harmonics energy in different ranges of XUV. The limits of HHG conversion efficiency may seem to have been attained in the cases of HHG in gases, plasmas, and solids under specular reflection from the solid surfaces. In most cases, they are less than 10^−6^ in the case of thirty orders of harmonics generated from surfaces [1,2], 10^−5^ in the cases of gases [3,4] and plasmas [5], and 10^−4^ in the case of resonance-enhanced single harmonics in plasmas [6]. These efficiencies are sufficient for attosecond pulse generation in the XUV range, although further enhancement allows improving the laser-matter interaction in this spectral range.

Among different methods of harmonic enhancement, one can admit the formation of the quasi-phase matching (QPM) conditions between the driving and generating waves during HHG [7,8,9]. This mechanism has been originally demonstrated in the visible range using solid materials [10]. Application of this concept in the case of the shorter-wavelength region requires conditions where the absorption of generating harmonic waves becomes insignificant with regard to the enhancement of this radiation in the conditions where the transfer of energy from driving to harmonic waves occurs at similar phase velocities. Since then, QPM has been demonstrated in gaseous [11,12,13,14] and plasma [15,16] media. One of the approaches here is the division of an extended medium into groups of smaller media separated by a vacuum. In these parts of the extended medium, the conditions of harmonics enhancement are maintained along the whole length (so-called coherence length) of divided plasmas and gas media until the phase difference Δ*φ* between converting and converted waves becomes equal to *π*.

In the case of gas HHG, the conditions of separation of the extended medium onto the group of smaller-sized media were realized by the formation of a few gas jets separated from each other by a distance equal to the sizes of these jets [12,13]. To form such jets, one has to drill the extended tube containing gas in a few places along the direction of femtosecond beam propagation. The obstacle of this method is the limitation of QPM conditions for a group of harmonics at the used intensities of the driving pulse.

HHG in laser-produced plasmas (LPP) allows the formation of variable conditions for QPM by using either extended perforated targets [17,18] or the application of multi-slit masks (MSM) placed in front of an ablating extended surface [19]. The manipulation of MSM geometry (i.e., sizes of slits, distance between slits, number of slits, tilting of MSM, etc.) allows the formation of the plasma jets of different spatial and concentration characteristics. Most important among them is the variation of free electron concentration in each of the jets of this multi-jet plasma (MJP) structure. The tuning of free electron concentration can be performed by changing the fluence of heating radiation on the target surface, as well as by tuning the pulse duration of heating radiation in a broad range. The former approach has been reported in previous studies [15,18], while the latter approach has not been realized. Notice that previous plasma QPM studies were carried out using the picosecond pulses as the heating radiation for the formation of the optimal plasma configurations dominantly containing atoms and ions.

In the meantime, the application of heating laser pulses for different durations and nanoparticle-containing targets can drastically change the characteristics of LPP. Those include the temperature of the plasma, ion and atom concentration, formation of clusters, quantum dots, nanoparticles (NPs) and microparticles (MPs), excitation of plasma, electron concentration, etc. Particularly, the electron concentration significantly depends on the temporal characteristics of the ablating beam. Thus, the studies of plasma QPM using the heating pulses of different durations in the case of nanoparticle-containing targets can clarify the methods for the creation of the “optimal plasma”, allowing the most efficient enhancement of the groups of harmonics in different ranges of XUV by combining the concepts of QPM- and NP-induced enhancement of HHG.

In this paper, we report our studies on the formation of QPM in different plasma plumes produced by nanosecond (ns), picosecond (ps), and femtosecond (fs) pulses on the surfaces of materials, allowing the formation of imperforated and perforated LPPs containing atoms, ions, NPs, and MPs. The optimization of QPM during HHG in such plasmas allowed the formation of the groups of harmonics with the enhancement factors exceeding 25×.

## 2. QPM Scheme for HHG in LPP

We used the 30 fs, 800 nm, 1 kHz, and 200 ps, 800 nm, 1 kHz pulses as the femtosecond and picosecond heating radiation from a Ti:Sapphire laser for plasma formation. The Nd:YAG laser (5 ns, 1064 nm, 10 Hz; Q-Smart, Coherent, USA) was synchronized with the Ti:Sapphire laser and used as a source of nanosecond heating radiation. The insignificant difference in the wavelengths of ns (1064 nm) and ps/fs (800 nm) pulses does not cause different dynamics in the absorbance, heating, melting, and evaporation of used samples. The most important difference in the ablation are attributed to different timescales of interaction of the heating pulses with the targets.

Both 30 fs and 200 ps pulses were generated from the same laser (Spitfire Ace; SpectraPhysics, Santa Clara, CA, USA). The 200 ps pulses were obtained by separation of the part of uncompressed radiation of this laser prior to entering the compressor stage. The delay between heating and driving pulses was adjusted by using the optical delay line for the latter pulses. The driving pulses were focused using a 400 mm focal length spherical lens in the plasma area, 0.3 mm above the target surface. The intensity of these pulses in the plasma area was 3 × 10^14^ W cm^−2^.

We used a new scheme of heating nanosecond pulses and driving femtosecond pulses from different laser sources for HHG in plasmas. An electronic delay between nanosecond and femtosecond pulses allowed applying sufficiently longer delays between two pulses compared with the optical delay technique. The delay between pulses was tuned using the delay generator (DG535; Stanford Research Systems, Sunnyvale, CA, USA). The synchronization of two laser sources, such as most commonly used Ti:Sapphire and Nd:YAG lasers, may resolve, to some extent, the puzzle related with the enhancement of harmonics in the multi-atomic particles produced during ablation of the bulk materials, or the targets initially containing these multi-atomic species. The main advantage of this approach is the control of the delay between the heating nanosecond pulses and the driving femtosecond pulses over a wide range between 0 and 10^5^ ns, which is assumed to be sufficient for analysis of the fast and slow components spreading out from the target in the laser-produced plasmas. The use of nanosecond Nd:YAG lasers as the sources of heating pulses may also offer some additional advantages compared with the commonly used picosecond pulses of the same repetition rate and wavelength as the driving sources. The application of nanosecond pulses to ablate the surface of targets allows the formation of less-ionized and less-excited plasma during longer periods of laser-matter interaction compared with picosecond pulses. This conclusion is based on the analysis of the nanosecond and picosecond ablation-induced plasma emission in the visible and XUV ranges in the case of formation of the “optimal” plasmas leading to generation of the highest harmonic yields. Notice that such plasmas formed by picosecond pulses have demonstrated stronger incoherent XUV emission. Additionally, Nd:YAG lasers commonly operating at a 10 Hz pulse repetition rate are more suitable for stable HHG in plasmas compared with the 1-kHz picosecond pulse ablation.

**Figure 1:**
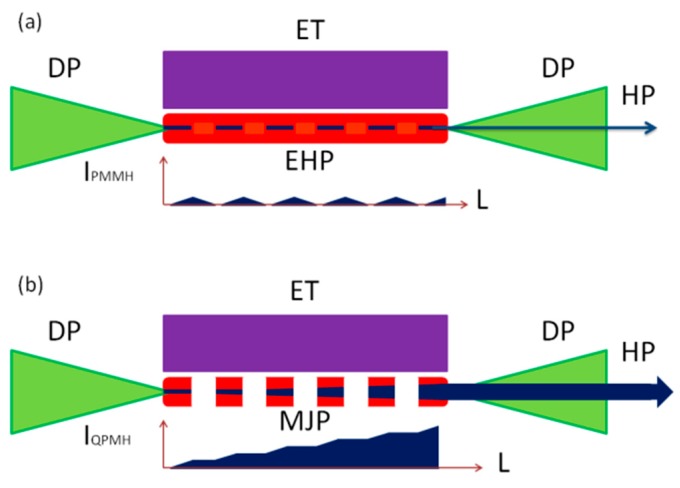
Schemes for non-quasi-phase matching (non-QPM) and QPM during high-order harmonic generation (HHG) in laser-produced plasmas (LPPs). (**a**) HHG in extended plasma. DP, driving femtosecond pulse; ET, extended target; EHP, extended homogeneous plasma; HP, harmonic pulse; *I*_PMMH_, intensity of phase mismatched harmonic; *L*, length of plasma. (**b**) HHG in multi-jet plasma. MJP, multi-jet plasma. *I*_QPMH_, intensity of the quasi-phase matched harmonic.

The heating pulses were focused using a 300-mm focal length cylindrical lens on the targets placed in the vacuum chamber to form the homogeneous extended plasmas (*l* = 5 mm, Figure 1a). The spatial characterization of spreading plasma has shown that it moves out from the ablating surface as a cone with a diverging angle of 20°. Thus, the spatial characteristics of plasma at the distance of 0.3 mm from the target were almost the same as the sizes of heating radiation on the ablating surface (~0.3 mm). The intensity of heating pulses on the target surface was varied up to 6 × 10^11^ W cm^−2^ (in the case of fs pulses), 4 × 10^9^ W cm^−2^ (in the case of ps pulses), and *I* = 1 × 10^9^ W cm^–2^ (in the case of ns pulses). One can see a significant difference in the optimal intensities of heating pulses on the target surface exceeding two and a half orders of magnitude. Meanwhile, the difference in the optimal fluencies of these pulses (0.2, 0.8, and 5 J cm^−2^ in the cases of fs, ps, and ns pulses) was notably smaller compared with the difference in their intensities. Our experiments showed that the maintenance of the fluence of heating pulses in this range allows the formation of QPM conditions.

To produce MJP (Figure 1b), the MSM was installed between the cylindrical lens and the target. We used three different MSMs to produce variable plasma jets on the 5-mm long target. Most of the experiments were carried out using the MSM comprising 0.2-mm slits separated from each other at the distance of 0.2 mm. We also used the MSMs comprising 0.5-mm slits separated from each other at a distance of 0.5 mm, and 0.8-mm slits separated from each other at a distance of 0.3 mm. The 50 ns delay was introduced prior to propagation of the 30 fs, 800 nm driving pulses through MJP. The maximal intensity of driving pulses in the plasma area was 5 × 10^14^ W cm^−2^. The generated harmonics were analyzed using home-made XUV spectrometer [19].

Boron NPs powder, silver MPs powder, and bulk silver sulfide were used as the targets. Boron NPs and silver MPs were purchased from Richest Group (Wuhan, China). The samples were provided in the form of powders. The mean sizes of B NPs were 50 nm, with the range of size distribution between 30 and 90 nm. The mean sizes of Ag MPs were 70 µm, with the range of size distribution between 50 and 90 µm. The NPs and MPs had spherical shapes. The powdered MPs, NPs, and mixtures of MPs and NPs were pressed to form tablets of 5-mm diameter using the electric hydraulic press machine (CY-24T; YKY, Shanghai, China).

We used silver sulfide as the target for ablation and QPM, since this plasma provides the generation of harmonics of 800 nm lasers up to the fifty-fifth order (H55, λ ≈ 15 nm). There are a few other species, such as silver, indium, and manganese, allowing generation of harmonics in this spectral range. These targets have already been studied in previous plasma QPM studies. The generation of as many harmonic orders as possible is a crucial requirement for observation and tuning of the group of enhanced harmonics in the 50–90 eV range. 

## 3. Results

Our first sample was the tablet containing powders of boron nanoparticles and silver microparticles (B NPs + Ag MPs). Both boron and silver plasmas that were ablated from bulk targets have shown harmonic generation greater than sixty orders [20,21]. One could expect the enhancement of harmonic yield from the plasmas containing nanoparticles and microparticles of these elements to behave similarly to previous studies of nanoparticle-containing plasmas [22]. Present studies confirmed that the conversion efficiencies of harmonics generated from these species were a few times larger than those from ablated bulk B and Ag. The reason for the application of the mixture of these two powders was to attempt to combine the attractive features of boron and silver emitters of harmonics; additionally, the formation of pressed tablets with suitable mechanical properties allowing the maintenance of surface properties during ablation by pulses of different durations (5 ns, 200 ps, and 30 fs).

**Figure 2:**
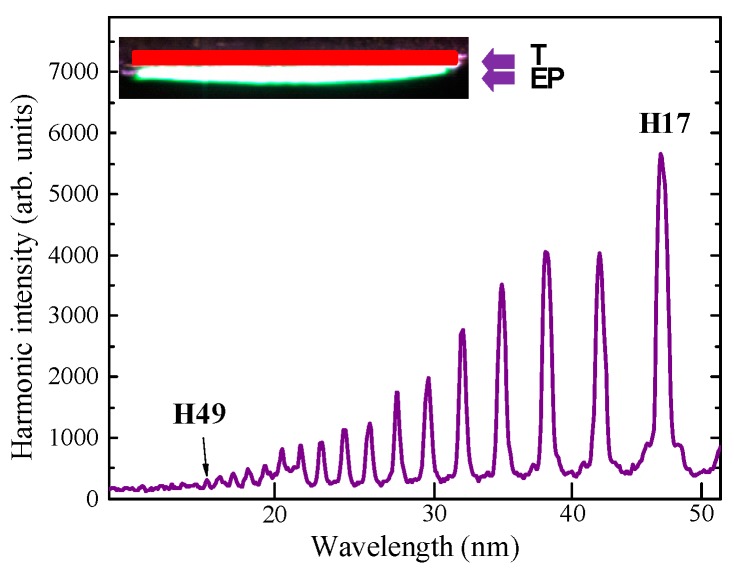
Harmonic spectrum produced from extended mixture of boron nanoparticles and silver microparticles (B NP + Ag MP) plasma. Inset: Image of extended plasma (EP) produced on the 5 mm long target (T) by focusing the 200 ps beam using a cylindrical lens.

Initially, we analyzed the harmonic generation in the extended plasmas using pulses of different durations. The envelopes of harmonic spectra in each of those cases had plateau-like shapes demonstrating the gradual decrease of each next harmonic order. Among three heating pulse regimes, fs and ps pulses provided better harmonic conversion efficiencies, while ns pulses allowed generation of weaker harmonics with the lower cutoff. The example of the harmonic spectrum from extended 5-mm plasma produced by 200 ps pulses on the surface of the B NP + Ag MP tablet is shown in Figure 2, while the shape of the extended plasma on the target surface is presented in the inset to this figure.

The spectrum of emitting radiation in the 15–50 nm range represented the set of harmonic orders, which were gradually decreased until the cut-off (H49). No significant enhancement of harmonics and cut-off extension were obtained with the gradual growth of the length of LPP. The reason for this restriction in the growth of harmonic yield is as follows. At some point in the generating medium, the phase difference between the harmonic that arrived at that point and the harmonic generated in that point reached *π*. Further increase of the phase difference led to destructive interference of harmonics up to phase difference of 2*π*. The harmonic yield increases and decreases along the whole length of the medium with the periodicity equal to the so-called coherence length (*L*_coh_). This pattern is illustrated in the *I*_PMMH_(*L*) curve (Figure 1a). One can admit that at these strongly phase-mismatched conditions, insignificant averaged accumulation of harmonic yield can be expected. Correspondingly, no enhancement of harmonic yield can be expected at these conditions in spite of the extended length of the nonlinear optical medium.

**Figure 3:**
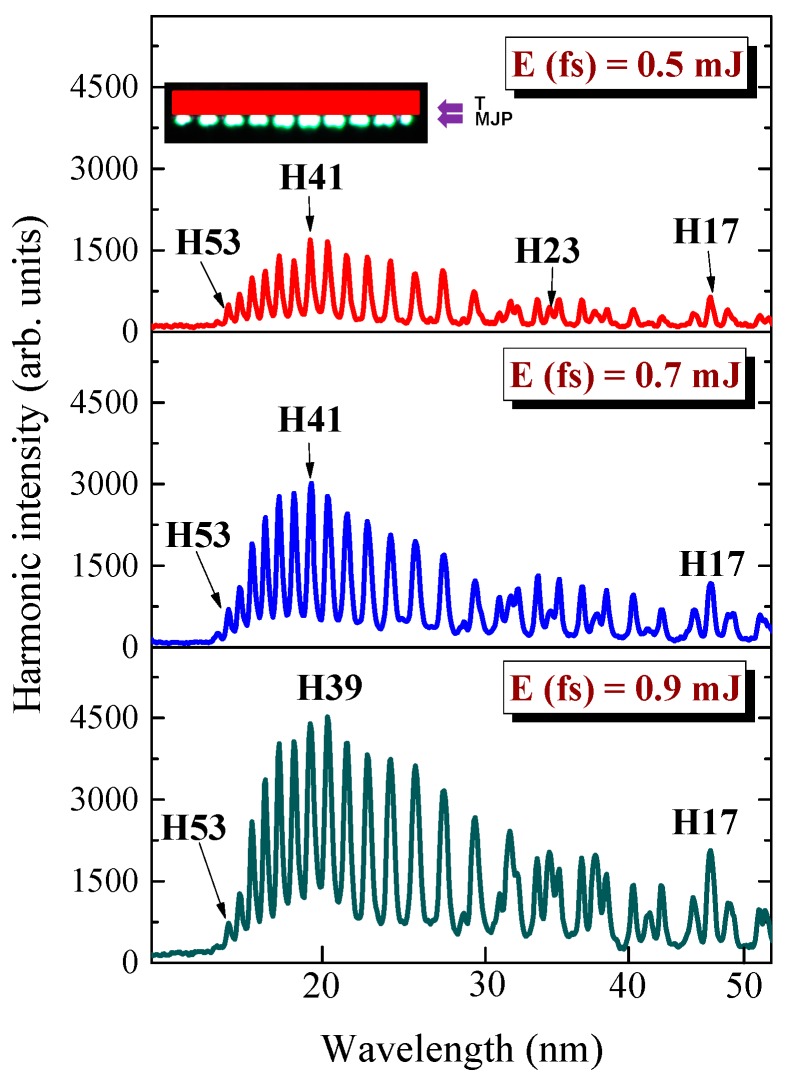
Harmonic spectra from perforated B NP + Ag MP LPP obtained at different energies of driving femtosecond pulses (0.5 mJ: upper panel; 0.7 mJ: middle panel; 0.9 mJ: bottom panel). Maximally enhanced QPM harmonic (H41 or H39) was maintained at these conditions. Inset: shape of micro-jet plasma (MJP) produced on the target surface.

Then we installed the MSM between the focusing cylindrical lens and target to produce the MJP containing ten 0.2-mm long jets separated from each other by 0.2 mm. The shape of this heterogeneous plasma formation is shown in the inset of Figure 3. The plasma was formed using fs, ps, or ns heating pulses. The whole length of these MJPs was two times smaller than the length of extended imperforated plasma.

Initially, we analyzed the QPM conditions in the case of MJP produced by fs pulses. We used different energies of driving fs pulses (0.5, 0.7, or 0.9 mJ). The 0.9 mJ heating fs pulses were used in each of these cases. The generated harmonic spectra at these three conditions of MJP formation are shown in Figure 3.

One can see a drastic change of the envelope of harmonic distribution obtained in the case of MJP (Figure 3) with regard to the case of extended imperforated plasma (Figure 2). Firstly, the harmonic yield in the longer-wavelength range was suppressed, while the shorter-wavelength harmonics became significantly enhanced. The enhanced harmonics were centered at around the thirty-ninth or forty-first orders (H39 or H41). These maximally-enhanced harmonics (*H*_qpm_) were almost the same in the case of three different energies of driving pulses, since the concentration of the free carriers responsible for QPM for some specific harmonic order was equal for the three regimes shown in Figure 3, due to same energy of heating fs pulses for the three shown cases. These experiments demonstrated that the role of free carriers appearing during propagation of driving femtosecond pulses through the plasma did not strongly influence the QPM conditions. The only difference in these three cases was the growth of the overall harmonic yield of QPM-enhanced harmonics and the broadening of the envelope of harmonic distribution centered at around *H*_qpm_. Notice a similarity in the ratio between the enhanced harmonics in the shorter-wavelength range and the suppressed harmonics in the longer-wavelength range.

Thus, in the conditions when the maintenance of the heating pulse’s energy did not significantly change the concentration of electrons, the maximal order of enhanced harmonics remained almost the same. Notice a significant decrease in the lower-order harmonics of these three spectra compared with the case shown in Figure 2, where the harmonics were generated in the extended imperforated plasma. The additional lines in the longer wavelength parts of spectra correspond to the second and third orders of diffraction of the enhanced groups of harmonics, apart from a few of them, which were attributed to the notably decreased lower-order harmonics (H17–H23).

QPM in LPP using spatial modulation of the medium is based on compensation of the phase mismatch Δ*φ* accumulated between the two fields with different frequencies (in HHG this is the driving field and its certain harmonics) when propagating in the dispersive medium. The commonly accepted understanding of optimal HHG QPM is that the multi-jet structure of plasma changes Δ*φ* from π at the exit from one jet to 2π at the beginning of another jet, so the separation between jets prohibits the destructive generation of harmonics with opposite directions of the electric field when π < Δφ < 2π. Notice that the transition from plasma jet to no-plasma interval is not as distinct, as, for example, the boundary between periodically poled crystal structures in classical QPM. The optical properties of no-plasma regions may also differ significantly from properties of plasma jets, so we consider the possibility for HHG QPM to be sub-optimal, fulfilling a less strict condition. Namely, the change of the phase at the exit of the jet is not necessarily π, but the total phase change of a harmonic after passing one jet and one no-plasma region is ≅2π, which one can call 2π QPM condition.

This 2π QPM condition is assumed to support the experimentally-observed near-quadratic growth of HHG efficiency with the number of jets. The enhancement of harmonic yield, in that case, is illustrated in the *I*_QPMH_(*L*) curve shown in Figure 1b. The yield of *H*_qpm_ gradually increases along the whole length of the perforated plasma, thus achieving significant enhancement. Notice that in the case of a single jet, when other slits in MSM were shielded, the envelope of the harmonic spectrum was similar to the one shown in Figure 2.

**Figure 4:**
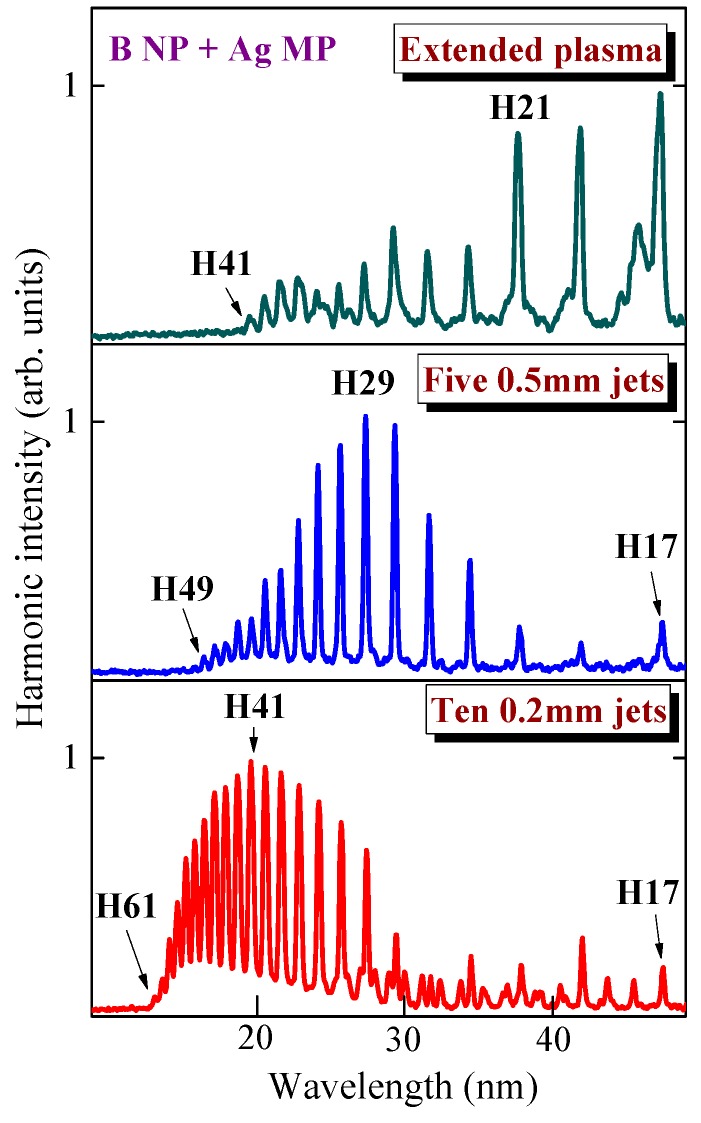
Normalized harmonic spectra from imperforated B NP + Ag MP LPP (upper panel), five 0.5 mm long jets (middle panel), and ten 0.2 mm long jets (bottom panel) obtained during ablation of the target by 200 ps pulses.

In the case of ps heating pulses, we observed an even better pattern of QPM for the narrower group of enhanced higher-order harmonics. Figure 4 shows the normalized spectra of the harmonics generated in B NP + Ag MP plasma. The upper panel corresponds to the extended imperforated plasma, and the two bottom panels correspond to the five 0.5-mm long lets and ten 0.2-mm long jets, respectively. The normalization of the intensity of the strongest harmonic to 1 allows the enhancement of the group of QPM harmonics to be clearly distinguished in different ranges of XUV with regard to the longer-wavelength region. The plateau-like harmonics in the case of 5-mm long plasma (upper panel) transform the group of enhanced harmonics at around of *H*_qpm_ = H25 in the case of five 0.5 mm long jets (middle panel). *H*_qpm_ then changed to H41 (i.e., similarly to the case of ablation by fs pulses) once we replaced this MSM with the one containing 0.2 mm slits (bottom panel).

The variation of *H*_qpm_ for different lengths of jets is attributed to the variation of *L*_coh_ of different harmonics. The larger *H*_qpm_ at the smaller length of the jet (*l*_jet_) should satisfy the optimal phase-matched conditions at a similar electron concentration in the plasma plume. The relation between these parameters should follow the *H*_qpm_ × *l*_jet_ ∞ const rule once a similar fluence of heating pulses irradiates the target [15,19]. This rule stipulates the decisive role of plasma dispersion over other mechanisms, leading to the variation of the difference between the phases of driving and harmonic waves. Notice that in the present case, this relation does not exactly satisfy the observation of the maximally enhanced harmonics (H29 and H41) for the two groups of plasma jets *l*_jet_ ratios, of which 0.5 mm/0.2 mm = 2.5. Different impeding mechanisms and conditions of experiments, such as heterogeneity of plasma jets and their concentrations, role of clusters of different sizes on the dispersion of plasma, etc., can cause the deviation from the above relation.

**Figure 5:**
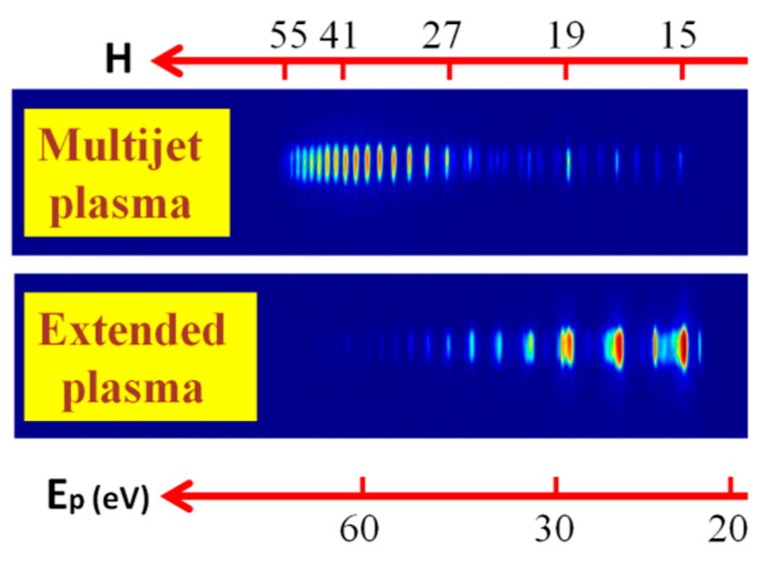
Images of harmonic distribution along the extreme ultraviolet (XUV) range in the case of silver sulfide plasma produced by picosecond (ps) pulses on the solid surface. Upper panel shows the case of 10-jet plasma obtained using the MSM comprising the 0.2 mm slits. Bottom panel shows the case of extended imperforated 5 mm long plasma. *H*, harmonic orders; *E*_p_(eV), energy of photons.

The comparison of the raw images of harmonics visually demonstrates the notable difference in the harmonic spectra produced in the imperforated and perforated plasmas. Figure 5 shows this comparison in the case of the plasma produced by picosecond pulses on the surface of silver sulfide in the cases of the absence (bottom panel) and presence (upper panel) of the MSM comprising the 0.2 mm slits. One can see a dramatic enhancement of higher orders and a significant suppression of the lower-order harmonics in the case of MJP with regard to extended plasma. Notice that higher harmonics (i.e., those above the 31st order) were barely seen in the case of extended plasma. The enhancement factor of H41 in the case of MJP was calculated to be 27×.

**Figure 6:**
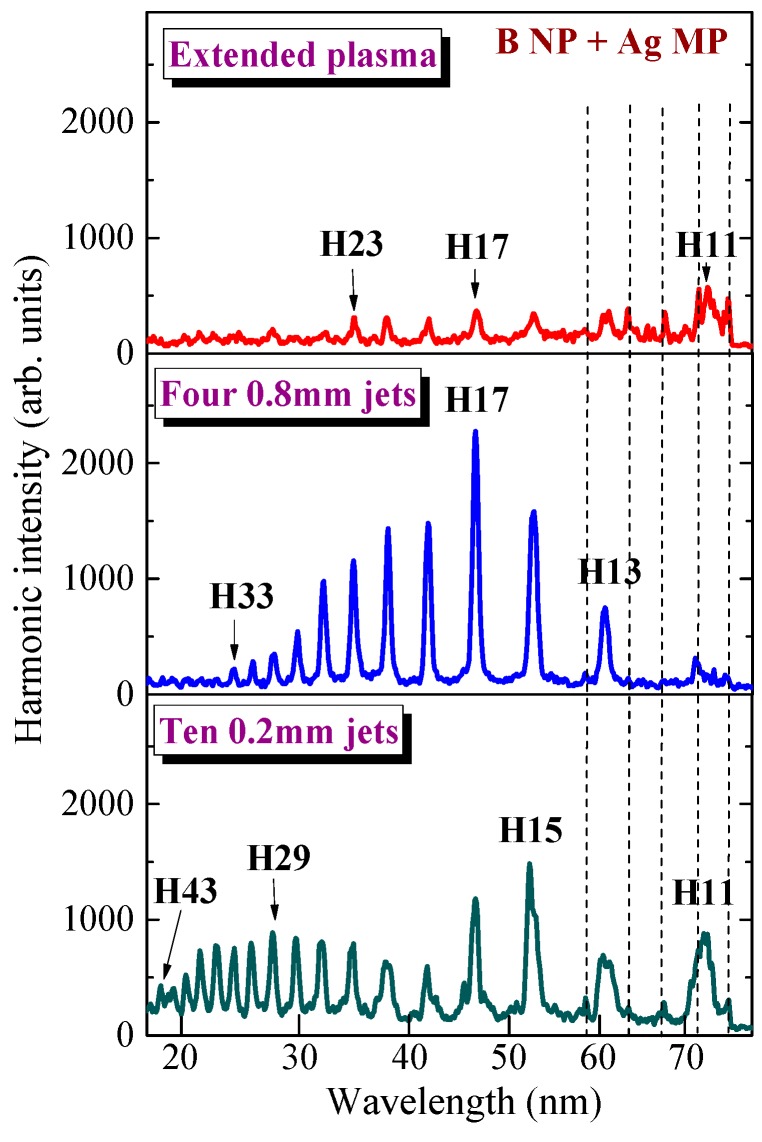
Harmonic spectra from imperforated B NP + Ag MP LPP (upper panel), four 0.8 mm long jets (middle panel), and ten 0.2 mm long jets (bottom panel) obtained during ablation of target by 5 ns pulses. Dashed lines show the plasma emission.

Quite another pattern appeared in the case of perforated plasma produced by ns pulses on the surface of the pressed tablet containing the mixture of Ag microparticle and B nanoparticle powders (Figure 6). The HHG efficiency and harmonic cut-off significantly decreased in the case of extended imperforated plasma produced by 5 ns pulses (Figure 6, upper panel) compared with the ps and fs cases. The perforation of plasma using the masks containing 0.2 and 0.8 mm slits cased the formation of QPM conditions at around H17 and H29, respectively (Figure 6, middle and bottom panels). One can see a lesser difference between the envelopes of these harmonic spectra compared with the cases of ablation by fs and ps pulses. The enhancement factors of *H*_qpm_ (H17 and H29) in the case of these two MJP configurations produced by ns pulses were smaller compared with those achieved during ablation by shorter pulses.

## 4. Discussion

The main goal of this work was the demonstration of the conditions when NP- and MP-contained plasmas satisfy the formation of QPM for different groups of harmonics. The presence of atoms of the same consistency, which obviously existed in LPP due to, for example, partial disintegration of ablating NPs and MPs, is less influential with regard to much denser low-dimensional species. We did not aim to compare the cases when plasma contained only atoms, or atoms + NPs or MPs. Previous studies revealed the fulfillment of QPM concept in the case of the presence of atoms and ions in the plasma area. Our studies, for the first time, have demonstrated that presence of molecular and low-dimensional species in plasma can also lead to formation of the QPM conditions.

In this paper, no variations of heating pulse energy on the surface of NP or MP target leading to variations in particle concentration were analyzed, since this issue has been earlier reported in previous studies [19,23,24]. Some indirect analysis of the influence of particle concentration on QPM was carried out by varying the fluence of heating pulses, either fs, or ps and ns. However, it is almost impossible to maintain the consistency of similar plasma conditions in these three cases. Notice that particle concentration is not a crucial issue in QPM concept. The most important component influencing the QPM conditions in LPPs is the concentration of electrons. To analyze this influence, one has to fix the concentration of harmonic emitters, otherwise it would be difficult to distinguish different processes affecting QPM in plasma.

The plasma at appropriate conditions of ablation of solid targets contains the nanoparticles aggregated from atoms. It has been confirmed during numerous studies of the debris deposited on nearby surface. Meanwhile, the formation of these NPs strongly depends on the experimental conditions, such as material of target, pulse duration, and fluence of heating radiation, etc. The analysis of the role of NPs and MPs in QPM becomes questionable once we ablate the bulk materials due to instability of the appearance of these low-dimensional structures in the area of driving pulse propagation. To resolve this problem, we prepared the targets, which have already contained these low-dimensional species, such as commercially available NPs and MPs, and then properly ablated those targets to form the LPPs containing these species. This concept allowed maintaining NPs and MPs in plasma without their drastic disintegration. The presence of these species in LPP has been proven by SEM analysis of the debris of ablated material containing approximately the same species with almost similar sizes as the NPs and MPs attached to the target.

Below we address the difference in the harmonic enhancement observed in our studies using variable conditions of plasma formation. Ablation by fs and ps pulses allowed the formation of relatively dense plasma (>5 × 10^17^ cm^−3^), while electron concentration was maintained at ~10% of the plasma concentration. The influence of these free electrons on the phase mismatch between interacting waves can be compensated for by formation of the multi-jet configuration of ablated material when the length of each plasma jet corresponded to *L*_coh_ of some higher-order harmonic order and a few surrounding harmonic orders. These QPM conditions allowed the generation of the group of enhanced harmonics in the XUV region. Another situation occurs in the case of target ablation by ns pulses. To achieve a similar concentration of plasma, one has to use stronger fluence, which causes the appearance of a notably larger amount of free electrons. These electrons significantly suppress the conversion efficiency of harmonics. At these conditions, the application of QPM concept did not allow harmonic enhancement similar to those attained during ablation of the target by shorter pulses.

The electron density in plasmas is one of the central issues in the QPM concept. Meanwhile, this method itself can provide the valuable information using the relation between the electron concentration in plasma, maximally enhanced harmonics in QPM conditions, the wavelength of the driving field, and the sizes of the single plasma jet in the MJP configuration. A previous study [23] has revealed that this method of determination of the electron concentration in the low-density, low-ionized laser-produced plasma using the nonlinear optical process of HHG of ultrashort pulses at the conditions of QPM of driving and harmonic waves is quite reliable and can be compared with the calculations of this parameter using the basic radiation hydrodynamics simulation code HYADES [25]. 

In our case, the electron density of used LPPs can also be defined using the QPM relation. The coherence length (measured in millimeters) at the conditions of using the 800-nm driving laser could be presented as *L*_coh_ ≈ 1.4 × 10^18^/*H*_qpm_ × *N*_e_ [26]. Here, *N*_e_ is the electron density in the plasma jets measured in cm^−3^. This simple formula allows definition of the electron density by knowing the coherence length, which is equal to the length of the single jet at the conditions of QPM in MJP, and the maximally enhanced harmonic order. The details of applicability of this formula are discussed in other studies [15,19,23]. In our case, the electron density in silver sulfide plasma was calculated to be 1.7 × 10^17^ cm^−3^ in the case of 0.2-mm-long jets and at the maximally enhanced harmonics centered at around the forty-first order (Figure 5).

Additional diagnostics are required to define the consistence of LPPs. Though it is extremely difficult to provide the time-of-flight mass spectroscopy measurements alongside the operating HHG facility, some estimates have been reported during previous studies of carbon-contained clusters, which revealed their presence in plasma at the moment of propagation of the driving wave [27,28]. Another diagnostic confirming the presence of low-dimensional species in the plasmas is based on the analysis of the debris of deposited material on the nearby substrates. The observation of low-dimensional species using SEM of these substrates confirmed the presence of boron NPs and silver MPs in the plasmas.

The simulations of QPM in plasmas has been reported in a few studies, thus providing different approaches in determination of the most important physical processes involved in artificially created conditions for maintenance of the optimal phase relations between the driving and harmonic waves [19,24,29,30]. Currently, the mechanism of QPM in LPP seems, to some extent, well determined. As with any new study, this research is mostly concentrated on the unexplored issues of the plasma QPM concept. In the meantime, the analysis of the intensity of harmonics as a function of the number of plasma jets has earlier provided a proof of the decisive role of QPM in the variations of the harmonics spectrum [15,19]. The intensity of *H*_qpm_ with the increase of the number of plasma jets (*n*) has grown, as *I*~*n*^2^ up to *n* = 4. Further addition of jets led to some deviation from quadratic dependence. The violation of equality of the conditions for each next added jet, non-ideal conditions of propagation through jets, and low-order nonlinear optical properties of plasma may change the optimal phase relations between driving and harmonic waves, which causes the deviation of *I*(*n*) dependence from the quadratic one with the growth of the number of jets. We did not carry out similar studies, since they have been already reported in previous studies.

## 5. Conclusions

Previous plasma QPM studies were carried out using the picosecond pulses as the heating radiation for the formation of the optimal plasma configurations dominantly containing atoms and ions of ablated metals (Ag, Mn, In). In the present study, we have demonstrated this process in the molecular (Ag_2_S), nanoparticle, and microparticle (B and Ag) plasmas. We have reported the analysis of various regimes of LPP formation for the creation of conditions for maximal QPM-induced enhancement of the groups of harmonics in the XUV region. Laser plasma was formed on the surfaces of NP- and MP-contained targets using ablation by nanosecond, picosecond, and femtosecond pulses. Different conditions of laser plasma formation (extended and perforated plasma) and variable concentrations of free electrons in these three cases of laser ablation led to modifications of QPM conditions. The QPM using femtosecond and picosecond heating pulses during HHG in such plasmas allowed the growth of conversion efficiency of the groups of harmonics with the enhancement factors exceeding 25× in different ranges of XUV, while nanosecond pulses produced MJP, which did not allow similar enhancement of higher-order harmonics.

## References

[1] Norreys P.A., Zepf M., Moustaizis S., Fews A.P., Zhang J., Lee P., Bakarezos M., Danson C.N., Dyson A., Gibbon P. (1996). Efficient extreme UV harmonics generated from picosecond laser pulse interactions with solid targets. Phys. Rev. Lett..

[2] Teubner U., Pretzler G., Schlegel T., Eidmann K., Förster E., Witte K. (2003). Anomalies in high-order harmonic generation at relativistic intensities. Phys. Rev. A.

[3] Pfeifer T., Walter D., Winterfeldt C., Spielmann C., Gerber G. (2005). Controlling the spectral shape of coherent soft X-rays. Appl. Phys. B.

[4] Tsakiris G.D., Eidmann K., Meyer-ter-Vehn J., Krausz F. (2006). Route to intense single attosecond pulses. New J. Phys..

[5] Ganeev R.A., Baba M., Suzuki M., Kuroda H. (2005). High-order harmonic generation from silver plasma. Phys. Lett. A.

[6] Ganeev R.A., Suzuki M., Baba M., Kuroda H., Ozaki T. (2006). Strong resonance enhancement of a single harmonic generated in extreme ultraviolet range. Opt. Lett..

[7] Paul A., Bartels R.A., Tobey R., Green H., Weiman S., Christov I.P., Murnane M.M., Kapteyn H.C., Backus S. (2003). Quasi-phase-matched generation of coherent extreme-ultraviolet light. Nature.

[8] Zhang X., Lytle A.L., Popmintchev T., Zhou X., Kaptayn H.C., Murnane M.M., Cohen O. (2007). Quasi-phase-matching and quantum-path control of high-harmonic generation using counterpropagating light. Nat. Phys..

[9] Bahabad A., Murnane M.M., Kapteyn H.C. (2010). Quasi-phase-matching of momentum and energy in nonlinear optical processes. Nat. Phys..

[10] Fejer M.M., Magel G.A., Jundt D.H., Byer R.L. (1992). Quasi-phase-matched second harmonic generation: Tuning and tolerances. IEEE J. Quantum Electron..

[11] Seres J., Yakovlev V.S., Seres E., Streli C.H., Wobrauschek P., Spielmann C.H., Krausz F. (2007). Coherent superposition of laser-driven soft-X-ray harmonics from successive sources. Nat. Phys..

[12] Pirri A., Corsi C., Bellini M. (2008). Enhancing the yield of high-order harmonics with an array of gas jets. Phys. Rev. A.

[13] Tosa V., Yakovlev V.S., Krausz F. (2008). Generation of tunable isolated attosecond pulses in multi-jet systems. New J. Phys..

[14] Fok T., Węgrzyński Ł., Kozlova M., Nejdl J., Wachulak P.W., Jarocki R., Bartnik A., Fiedorovicz H. (2014). High-order harmonic generation using a multi-jet gas puff target. Photonics Lett. Pol..

[15] Ganeev R.A., Suzuki M., Kuroda H. (2014). Quasi-phase-matching of high-order harmonics in multiple plasma jets. Phys. Rev. A.

[16] Wöstmann M., Splitthoff L., Zacharias H. (2018). Control of quasi-phase-matching of high-harmonics in a spatially structured plasma. Opt. Express.

[17] Ganeev R.A., Suzuki M., Kuroda H. (2014). High-order harmonic enhancement using the quasi-phase-matching in laser plasma. JETP Lett..

[18] Ganeev R.A., Suzuki M., Yoneya S., Kuroda H. (2014). Quasi-phase-matching induced enhancement of the groups of high-order harmonics generating in various multi-jet plasmas produced using perforated targets and modulated heating pulses. Laser Phys..

[19] Ganeev R.A., Tosa V., Kovács K., Suzuki M., Yoneya S., Kuroda H. (2015). Influence of ablated and tunneled electrons on the quasi-phase-matched high-order harmonic generation in laser-produced plasma. Phys. Rev. A.

[20] Ganeev R., Suzuki M., Baba M., Kuroda H., Ozaki T. (2005). High-order harmonic generation from boron plasma in the extreme-ultraviolet range. Opt. Lett..

[21] Elouga Bom L.B., Kieffer J.-C., Ganeev R.A., Suzuki M., Kuroda H., Ozaki T. (2007). Influence of the main pulse and prepulse intensity on high-order harmonic generation in silver plasma ablation. Phys. Rev. A.

[22] Singhal H., Ganeev R.A., Naik P.A., Srivastava A.K., Singh A., Chari R., Khan R.A., Chakera J.A., Gupta P.D. (2010). Study of high-order harmonic generation from nanoparticles. J. Phys. B.

[23] Ganeev R.A., Suzuki M., Yoneya S., Kuroda H. (2015). Electron density measurements using high-order harmonic generation in laser-produced plasmas. Appl. Phys. B.

[24] Ganeev R.A., Husakou A., Suzuki M., Kuroda H. (2016). Application of mid-infrared pulses for quasi-phase-matching of high-order harmonics in silver plasma. Opt. Express.

[25] Rubenchik A.M., Feit M.D., Perry M.D., Larsen J.T. (1998). Numerical simulation of ultra-short laser pulse energy deposition and bulk transport for material processing. Appl. Surf. Sci..

[26] Kan C., Burnett N.H., Capjack C.E., Rankin R. (1997). Coherent XUV generation from gases ionized by several cycle optical pulses. Phys. Rev. Lett..

[27] Lopez-Quintas I., Oujja M., Sanz M., Benitez-Cañete A., Hutchison C., de Nalda R., Martin M., Ganeev R.A., Marangos J.P., Castillejo M. (2014). Characterization of laser-induced plasmas of nucleobases: Uracil and thymine. Appl. Surf. Sci..

[28] Ganeev R.A., Baba M., Suzuki M., Kuroda H. (2014). Application of extended carbon-based clustered plasma plumes for the high-order harmonic generation of ultrashort pulses. J. Appl. Phys..

[29] Strelkov V.V., Ganeev R.A. (2017). Quasi-phase-matching of high-order harmonics in plasma plumes: Theory and experiment. Opt. Express.

[30] Redkin P.V., Ganeev R.A., Guo C. (2019). Analytical treatment of quasi-phase matching in multijet laser plasmas: Influence of free electrons between jets, intrinsic phase, Gouy phase, and driving pulse’s group velocity. J. Phys. B.

